# Eye movement verification and evaluation of initial sandplay picture system for internet addiction symptoms in Chinese adolescents

**DOI:** 10.1186/s13052-023-01487-8

**Published:** 2023-07-16

**Authors:** Ying Ge, Jun Yu Huo, Hai Bo Yang, Jay L. Wenger, Jing Yi Yuan, Xiao Jie Sun

**Affiliations:** 1 Key Laboratory of Emotion and Mental Health in Chongqing, School of Education, Chongqing University of Arts and Sciences, Chongqing, 402160 China; 2 School of Humanities & Social Sciences, Fuzhou University, Fuzhou, 350116 China; 3 College of transportation engineering, Tongji University, Shanghai, 201804 China; 4 Academy of Psychology and Behavior, Tianjin Normal University, Tianjin, 300387 China; 5 Social Sciences Division, Harrisburg Area Community College, Central Pennsylvania’s Community College, Lancaster, PA 17602 USA; 6 Chongqing Qijiang District Special Education School, Chongqing, 401420 China

**Keywords:** Chinese internet-addicted adolescents, Symptoms, Initial sandplay picture system, Eye movement verification

## Abstract

**Background:**

Sandplay therapy is a psychotherapeutic technique, based on the psychoanalytic theory of the unconscious. Nearly a century after it was developed, sandplay can now be applied for the initial diagnosis tools for sand players. The goal of the current research is to demonstrate the role of sandplay in identifying internet-addicted adolescents in China. The study aims to evaluate the reliability and validity for sandplay as a diagnosis and evaluation tool for internet addiction symptoms, and to verify the consistency that exists between results based on sandplay pictures and those based on the Pathological Internet Usage Scale for Adolescents (APIUS).

**Methods:**

The research was conducted with a 2 × 2 mixed factorial design – two types of participants (addicts and non-addicts) and two types of sandplay pictures (pictures for addicts and pictures for non-addicts). An absolute recognition-judgment paradigm was used along with eye movement evaluations to evaluate the existing initial sandplay picture system for internet addiction symptoms (22 sandplay pictures, 11 related to addicts and 11 related to non-addicts, respectively). Sixty Chinese adolescents were selected as the participants (30 as addicts and 30 as non-addicts) according to the APIUS.

**Results:**

(1) The initial sandplay pictures for internet addicts are clearly preferred by Chinese internet-addicted adolescents, which are more familiar and easier to process; (2) Such pictures have a higher level of emotional arousal and cognitive resonance for the addicts; (3) Track and heat maps indicate that young internet addicts mainly fixate on the initial sandplay pictures for internet addicts.

**Conclusion:**

This initial sandplay picture system can be used to screen and identify young Chinese internet addicts based on symptoms, and the evaluation results are consistent with those based on the APIUS.

**Supplementary Information:**

The online version contains supplementary material available at 10.1186/s13052-023-01487-8.

## Introduction

In the course of rapid network development, the internet has become a daily necessity for the general public. While it facilitates our life, it also has harmful effects. Adolescents lacking self-control and discernment can easily become addicted to the internet and what it affords. Goldberg [1] first introduced the concept of internet addiction, and defined it as a maladaptive pattern of internet use leading to clinically-significant social and psychological impairment and distress. Young [2] then studied internet addiction comprehensively. At present, the term “internet gaming disorder” has been officially included in *The Diagnostic and Statistical Manual of Mental Disorders* (DSM-5). It is defined as a mental and behavioral disorder caused by excessive internet use – characterized by a strong desire to use the internet along with withdrawal symptoms after reduction or cessation of internet use. It can cause mental and somatic symptoms [3].

In China, 80%~90% of internet addicts are gaming addicts. Specifically, online gaming has become an important practice for Chinese adolescents [4, 5]. The meaning of internet addiction can be derived from the description above. Even though internet addiction has not yet been explicitly included in the current DSM, the definition of internet gaming disorder is used in research about internet addiction.

As a social hotspot, adolescents’ internet addiction is prominent in all sectors of society. In recent years, researchers have explored the causes and characteristics of adolescents’ internet addiction in terms of social relations and family education, using a variety of methodologies and interventions. Some researchers have used structured questionnaires [6, 7]. Other researchers have explored the neural mechanisms, attentional biases, and other psychological characteristics of internet addicts with experimental methods [8–10]. And some have provided therapeutic interventions, such as group therapy, family therapy, and individual therapy, for adolescent internet addicts [11, 12]. Furthermore, researchers are committed to the development, modification, and application of test instruments for internet addiction, with the aim to establish highly standardized and objective instruments. For now, these include structured questionnaires and scales [2, 13–15]. Few researchers have tried projection tools to identify adolescent internet addicts and then evaluated the objectivity of such tools.

Sandplay therapy is a psychotherapeutic technique, developed by Jungian analytical psychologist Doar Kalff, and based on the psychoanalytic theory of the unconscious [16]. It has wide international influence. With the freedom and protection created by sandplay therapists, sandplayers can use a sandbox with sand, water and sand tools to manifest their own intangible psychological contents in an appropriate symbolic way. They can also express their own prelinguistic experience and blocked mental energy, thus gaining a more integrated development [17].

Nearly a century after it was developed, sandplay therapy was introduced to China in the 1990s [18]. It can now be applied for diagnoses and treatments. Specifically, sandplay can be used as an indicator for the initial diagnosis tools for sand players [19]. Empirical studies are intended to seek objective support for this technique by verifying its standardization and effectiveness. Aoki [20] studied the retest reliability of the sandplay technique, demonstrating the difference in emotion regulation between a juvenile delinquent group and the normal group. Fan et al. [21] verified the effectiveness of sandplay therapy in psychotherapy for young children through a combination of questionnaires and interviews. During a therapeutic intervention, the initial sandplay is the first model made by a sand player. It indicates the relationship between the player’s consciousness and unconsciousness, and it can indicate the essence of personal problems and provide the sandplayer with clues and directions to solve problems [16, 17, 22].

At present, sandplay-based research on internet addiction mainly focuses on therapeutic intervention [23–25]. Although Ge et al. [26] explored the initial sandplay characteristics of adolescent internet addicts in China and established a standardized initial sandplay picture system for Chinese adolescents’ internet addiction symptoms [27]. But the objectivity of sandplay as a diagnosis and evaluation tool for internet addiction symptoms has not yet been verified.

Eye movement research involves recording real-time data as research participants view visual information. Through eye-movement tracking, researchers can explore the cognitive and emotional processing of internet addicts [28–30]. Eye movements tracking can be a useful, objective strategy for evaluating participants. Researchers have used eye movements to evaluate the objectivity of projective tests – for example, the Rorschach Inkblot Method (RIM) [31, 32] and the Thematic Apperception Test (TAT) [33]. The eye-movement indicators of different subject groups during these tests (RIM and TAT) allowed researchers to identify important differences between the groups, thus providing objective support for the tests.

In sum, internet addiction is an important concern. It is a behavioral addiction that involves complex physical and psychological factors. Thus, various methods should be used to evaluate it. We believe sandplay presents a new and potentially fruitful way to measure internet addiction. We base the current research on the absolute recognition-judgment paradigm (showing the holistic recognition ability of individuals) [29, 30, 34], and specifically we used an SMI eye tracker to track and record the real-time eye movement indicators of adolescent internet addicts and non-addicts, during their recognition of initial sandplay pictures. Based on these indicators, as well as eye movement track maps and heat maps, we further verify the objectivity of the initial sandplay picture system for Chinese adolescents’ internet addiction symptoms. Also, we evaluated the function of the initial sandplay in identification and diagnosis of internet addiction symptom from the perspective of ecological validity. Finally, we verified the consistency between the evaluation results based on sandplay pictures and those based on the Pathological Internet Usage Scale for Adolescents (APIUS).

## Methods

### Participants

Junior high school students from Chongqing, China were selected to complete the Adolescent Pathological Internet Use Scale (APIUS) prepared by Lei et al. [14]. The questionnaire includes 38 items in 6 dimensions including salience, social comfort, tolerance, compulsive internet use, withdrawal symptoms, and negative outcomes. It is based on a 5-point system ranging from 1 (i.e., completely inconsistent) to 5 (i.e., completely consistent). APIUS has been shown to have good psychometric properties among Chinese adolescent subjects [5–47]. In the research, coefficient α was taken as 0.948, with relatively high reliability and validity. The questionnaires were then collected for data analysis. The students with an average score greater than or equal to 3.0 points were classified as internet addicts, and those with an average score less than 3.0 points as non-addicts. Overall, 60 Chinese adolescents were selected as participants (30 as addicts and 30 as non-addicts), including 28 boys and 32 girls, with their age ranging from 13 years to 14 years, and their net age of more than 2 years. The researchers then conducted Fisher’s exact test and independent sample *t*-test. The test results showed that there was no statistically significant difference in gender between the two groups (*P* > 0.05) and no statistically significant difference in age between the two subject groups (*t* = 1.454, *P* > 0.05).

### Instruments

An SMI eye tracker (with sampling frequency of 500 Hz) was connected to two host computers. One host computer was equipped with SMI Experiment Center 2.0 for stimulus presentation, and the other was the ivies host computer for calibration, and recording and storage of eye-movement data. With SMI Experiment Center 2.0, the researchers edited the experimental program (including pictures and instruction required for the experiment), realized synchronous presentation and switching of stimuli between the two host computers by enabling the communication port COM1, and cut the eye-movement data of different stimuli to form a complete set. The two host computers transmitted data through the communication port COM1 at a baud rate of 9,600 bits per second, and 8 data bits.

### Experimental design and procedure

The research was conducted with a 2 × 2 mixed factorial design -- two types of participants (addicts and non-addicts) and two types of sandplay pictures (pictures for addicts and pictures for non-addicts). In the research, the participant type and the picture type were selected as independent variables, and the eye movement indicators (fixation duration, fixation count, number of saccades, and latency of saccades) and behavioral indicators served as dependent variables.

Before the formal experiment, a preliminary experiment was conducted on 5 participants with the same procedure as the formal one. The procedures were sound, and the results were trustworthy.

First, we let participants sit down, and then we carried out calibrations before starting the experiment. In the experiment, a 13-point calibration was carried out using the view program provided by the Hi-Speed eye tracker. The participants were required to look at the 13 dots randomly presented on the display, one by one. The experiment could only be started when the calibration results met the requirements that both X and Y are less than or equal to 2°. During the formal experiment, experimental instructions were presented on the computer. The experimenter then explained the instructions to the participants as follows: Press F if you like the picture, and press J if you don’t like the picture.

Second, we started the exercise. Five sandplay pictures were presented randomly, each for a period of 3,500 ms. The participants pressed F or J on the keyboard according to their preference, and then the corresponding picture disappeared. If no response was received within 3,500 ms, the trial would not be recorded.

**Fig. 1:**
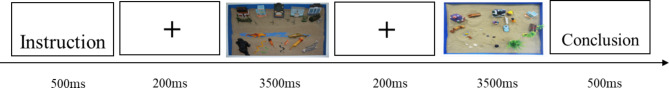
Flow Chart of Eye Movement Test

Finally, we started the formal experiment. Twenty sandplay pictures were presented randomly, each for a period of 3,500 ms. The participants pressed F or J according to their first impression of the corresponding picture. and then the corresponding picture disappeared. If no response was received within 3,500 ms, the trial would not be recorded. Refer to Fig. 1 for the procedure.

### Experimental materials

Pictures used in the experiment were selected from the initial sandplay picture system for Chinese adolescents’ internet addiction symptom established by Ge et al. [27]. There are 22 pictures in total (11 for addicts and 11 for non-addicts), each having a size of 19.6 × 11.4 cm. The pictures for addicts contained more military conflicting sand tools, and few sand tools featuring daily life and nature. Their themes were dominated by traumatic division and confusion other than integration and penetration, while the pictures for non-addicts were exactly the opposite.

### Data processing

The SPSS20.0 statistical software was used for data processing. Before analysis and processing findings, inappropriate data were deleted. This included invalid data and eye-movement data that could not be recorded by the eye tracker, due to a participant’s head movement, failure to keep eyes on the screen, or other physiological reasons. Effect sizes and statistical power were analyzed using Gpower3.1.9 0.7.

## Results

The dependent variable indicators included behavioral indicators (response time, and preference selection) and eye-movement indicators (fixation duration, fixation count, number of saccades, and latency of saccades).

### Behavioral data

#### Response time

In terms of response time, the interaction of participant type and picture type was insignificant, with *F*_(1,57)_ = 0.963, *p* = 0.331, *η*_*p*_^*2*^ = 0.017, and statistical power of test = 0.517. At the same time, there were insignificant main effects of participant type (*F*_(1,57)_ = 1.747, *p* = 0.192, *η*_*p*_^*2*^ = 0.030, statistical power of test = 0.907) and picture type (*F*_(1,57)_ = 2.997, *p* = 0.090, *η*_*p*_^*2*^ = 0.050, statistical power of test = 0.938). Refer to Table 1 for details.

**Table 1:** Behavioral Data for Absolute Recognition-Judgment of Eye Movements

Group	Picture Type	Preference Selection (M ± SD)	Response Time (M ± SD)
Addict group	Sandplay pictures for addicts	3.58 ± 0.17	1487.75 ± 636.98
	Sandplay pictures for non-addicts	3.45 ± 0.20	1456.92 ± 579.03
Non-addict group	Sandplay pictures for addicts	3.50 ± 0.21	1348.15 ± 531.91
	Sandplay pictures for non-addicts	3.55 ± 0.21	1235.99 ± 411.76

#### Preference selection

In terms of preference selection, the interaction of participant type and picture type was significant, with *F*_(1,57)_ = 4.999, *p* = 0.029, *η*_*p*_^*2*^ = 0.081, and statistical power of test = 1.000. After a simple effect test, it was found that there was a significant difference in the preference for the two types of pictures among the internet addicts, with *F*_(1,57)_ = 4.960, *p* = 0.030, *η*_*p*_^*2*^ = 0.023, and statistical power of test = 0.805. Furthermore, the sandplay pictures for internet addicts were clearly preferred (Table 1).

### Eye movement indicator data

Refer to Tables 2 and 3 for the descriptive statistical analysis and multi-factor analysis of variance of eye movement indicators (fixation duration, fixation count, number of saccades, and latency of saccades).

**Table 2:** Description Results for Absolute Recognition-Judgment of Eye Movements

Participant Type	Picture Type	Fixation Duration	Fixation Count	Number of Saccades	Latency of Saccades
Addict group	Sandplay pictures for addicts	248.54 ± 234.34	12.03 ± 2.42	12.31 ± 4.67	219.06 ± 83.60
	Sandplay pictures for non-addicts	305.08 ± 222.93	10.71 ± 2.66	11.29 ± 4.46	238.20 ± 19.08
Non-addict group	Sandplay pictures for addicts	302.97 ± 355.75	10.56 ± 1.93	9.89 ± 2.30	306.13 ± 105.00
	Sandplay pictures for non-addicts	294.51 ± 238.57	10.66 ± 2.17	10.46 ± 2.60	275.12 ± 71.32

**Table 3:** Multi-factor Analysis of Variance for Absolute Recognition-Judgment of Eye Movements

Independent Variable	Fixation Duration	Fixation Count	Number of Saccades	Latency of Saccades
Participant Type	3.489	10.879	3.489	2.367
Picture Type	0.350	3.185	0.350	0.624
Participant type * picture type	2.240	4.397*	4.479*	4.332*
*η*_*p*_^*2*^	0.038	0.072	0.073	0.071
Power of test	0.857	0.989	0.987	0.987

Note: *p < 0.05

#### Fixation duration

In terms of fixation duration, the multi-factor analysis of variance showed that the interaction of participant type and picture type was insignificant, with *F*_(1,57)_ = 2.240, *p* = 0.140, *η*_*p*_^*2*^ = 0.038, and statistical power of test = 0.857. At the same time, the main effects of participant type (*F*_(1,57)_ = 3.489, *p* = 0.718, *η*_*p*_^*2*^ = 0.002, and statistical power of test = 0.857) and picture type (*F*_(1,57)_ = 0.350, *p* = 0.317, *η*_*p*_^*2*^ = 0.018, and statistical power of test = 0.950) were also insignificant. Refer to Table 3 for details.

#### Fixation count

In terms of the fixation count, the multi-factor analysis of variance showed that the interaction of participant type and picture type was significant, with *F*_(1,57)_ = 4.397, *p* = 0.040, *η*_*p*_^*2*^ = 0.072, and statistical power of test = 0.989. Refer to Table 3 for details. After a simple effect test, it was found that the fixation count on sandplay pictures by addicts was greater than that of sandplay pictures by non-addicts (with *F*_(1,57)_ = 6.714, *p* = 0.012, *η*_*p*_^*2*^ = 0.105, and statistical power of test = 1.000). Among the addicts, the fixation count of sandplay pictures for addicts was greater than that of sandplay pictures for non-addicts (with *F*_(1,57)_ = 7.408, *p* = 0.009, *η*_*p*_^*2*^ = 0.115, and statistical power of test = 0.999).

#### Number of saccades

In terms of the number of saccades, the multi-factor analysis of variance showed that the interaction of participant type and picture type was significant, with *F*_(1,57)_ = 4.479, *p* = 0.039, *η*_*p*_^*2*^ = 0.073, and statistical power of test = 0.987 (Table 3). After a simple effect test, it was found that the number of saccades on sandplay pictures for addicts was greater than that of non-addicts (with *F* (_1,57_) = 6.464, *p* = 0.014, *η*_*p*_^*2*^ = 0.102, statistical power of test = 0.999). Among the addicts, the number of saccades on sandplay pictures for addicts was greater than that on the sandplay pictures for non-addicts (with *F*_(1,57)_ = 3.604, *p* = 0.063, *η*_*p*_^*2*^ = 0.059, and statistical power of test = 0.968).

#### Latency of saccades

With respect to the latency of saccades, the multi-factor analysis of variance showed that the interaction of participant type and picture type was significant, with *F*_(1,57)_ = 4.332, *p* = 0.042, *η*_*p*_^*2*^ = 0.071, and statistical power of test = 0.987 (Table 3). Refer to Table 3 for details. After a simple effect test, it was found that the latency of saccades on the sandplay pictures for addicts was lower than that of non-addicts, with *F*_(1,57)_ = 12.36, *p* = 0.001, *η*_*p*_^*2*^ = 0.178, and statistical power of test = 0.999.

### Eye movement track maps results

**Fig. 2:**
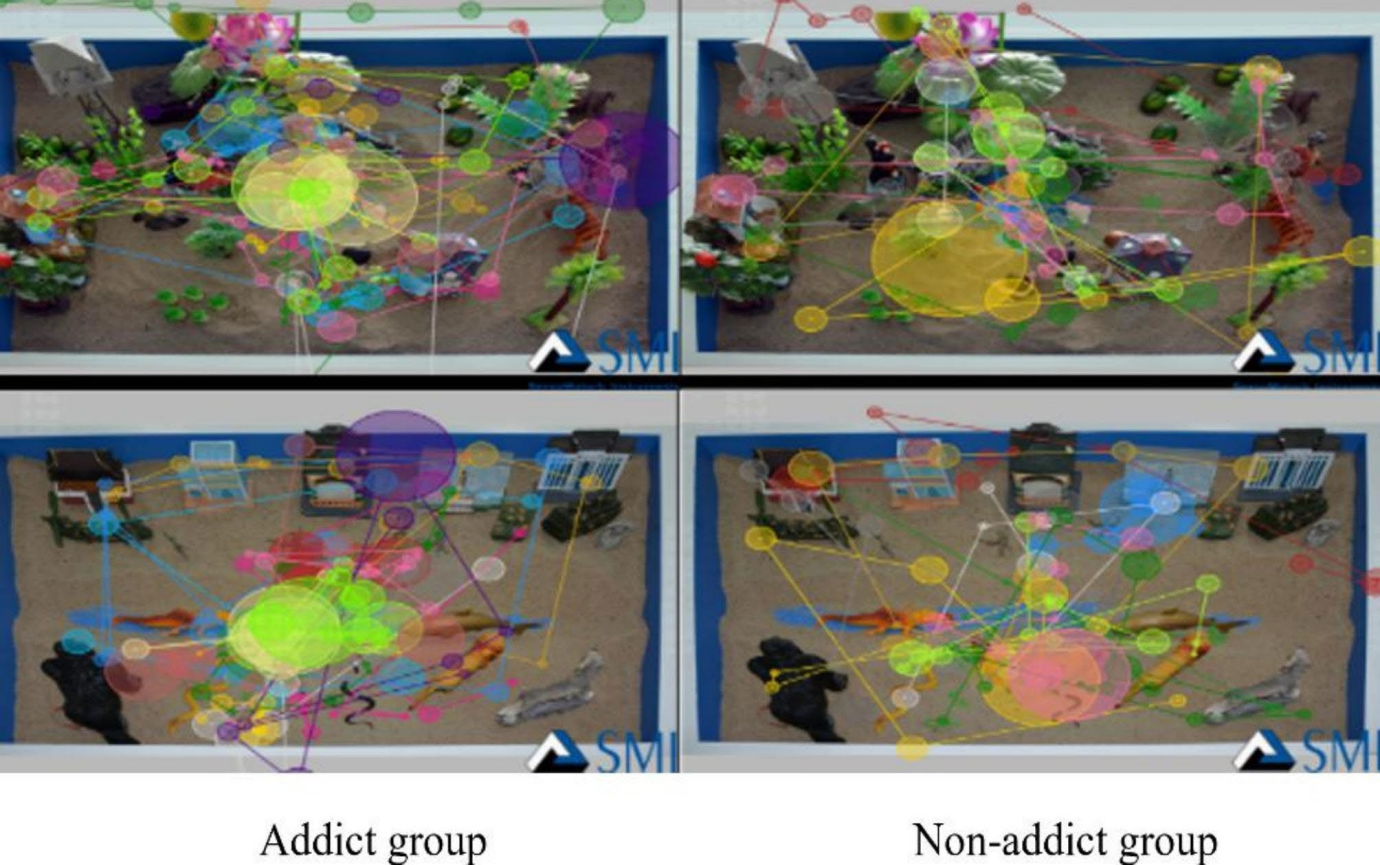
Eye Movement Track Maps of Two Groups during Fixation on Initial Sandplay Pictures for Internet Addicts

The eye movement tracks during fixation on the initial sandplay pictures for internet addicts are depicted in Fig. 2. As shown, there was a significant difference in the number of fixations between the two groups. To be specific, the fixation count on the sandplay pictures for addicts was obviously greater than that of the non-addict group. At the same time, for the addict group, the range of fixation was wider, the content was more comprehensive, and the fixations fo cused more on conflicts and confrontations in the pictures.

**Fig. 3:**
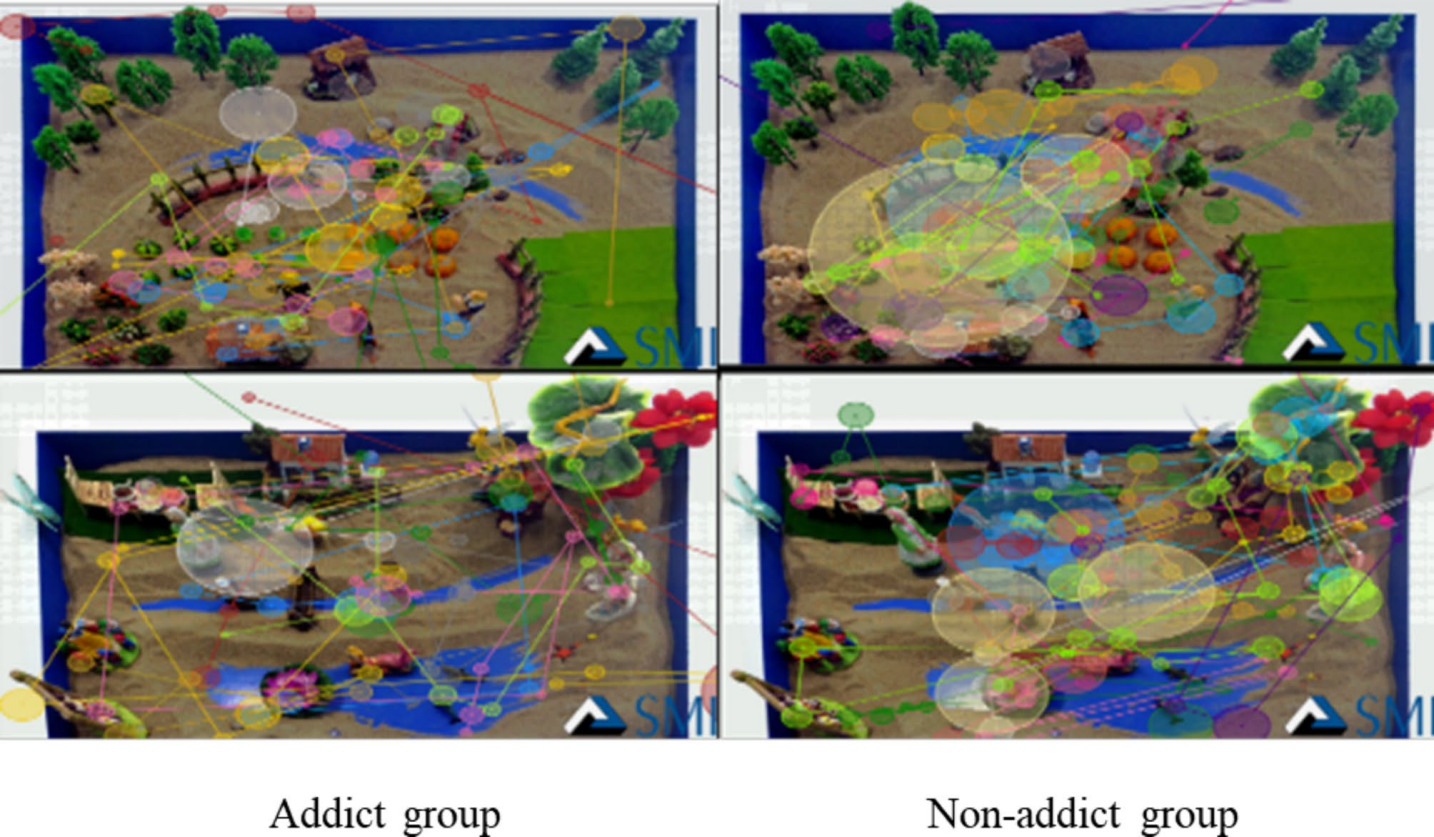
Eye Movement Track Maps of Two Groups during Fixation on Initial Sandplay Pictures for Non-Internet Addicts

As shown in Fig. 3, there was an insignificant difference in the number of fixations on the initial sandplay pictures for non-internet addicts between the two groups. However, the non-addict group had more fixations on the sandplay pictures for non-addicts and relatively small saccade amplitude, while the addict group had a few long-time fixations, and relatively large saccade amplitude.

### Eye movement heat maps results

**Fig. 4:**
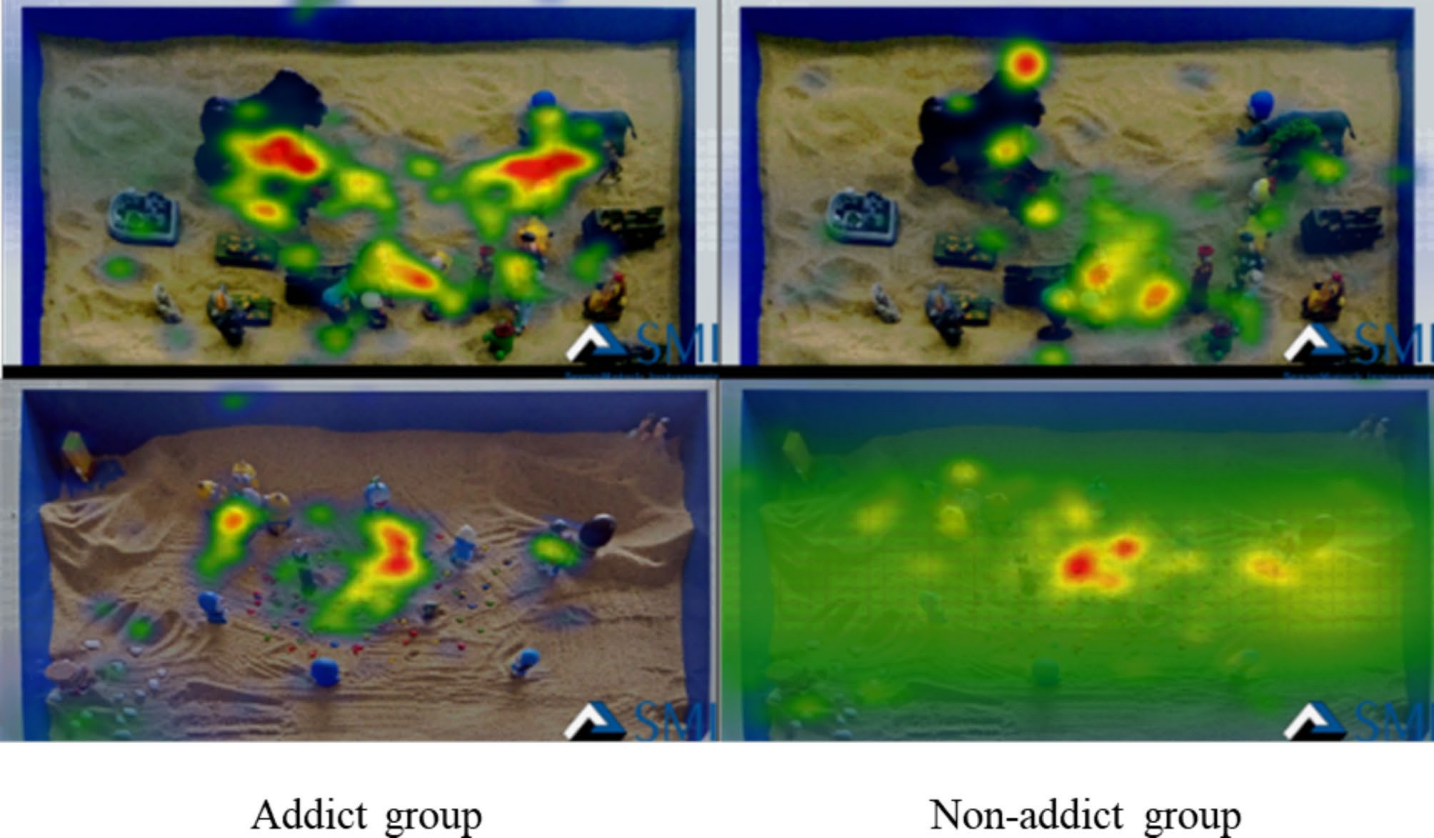
Eye Movement Heat Maps of Two Groups during Fixation on Initial Sandplay Pictures for Internet Addicts

The heat maps in the fixation process show the areas with high attention and the largest number of fixations by the participants. As shown in Fig. 4, there is a significant difference in the eye movement heat maps between the two groups during fixation on the initial sandplay pictures for internet addicts. Compared to the non-addict group, the addict group was more concentrated in the content of fixation, and paid more attention to conflicts, violence, threats and isolation in the pictures.

**Fig. 5:**
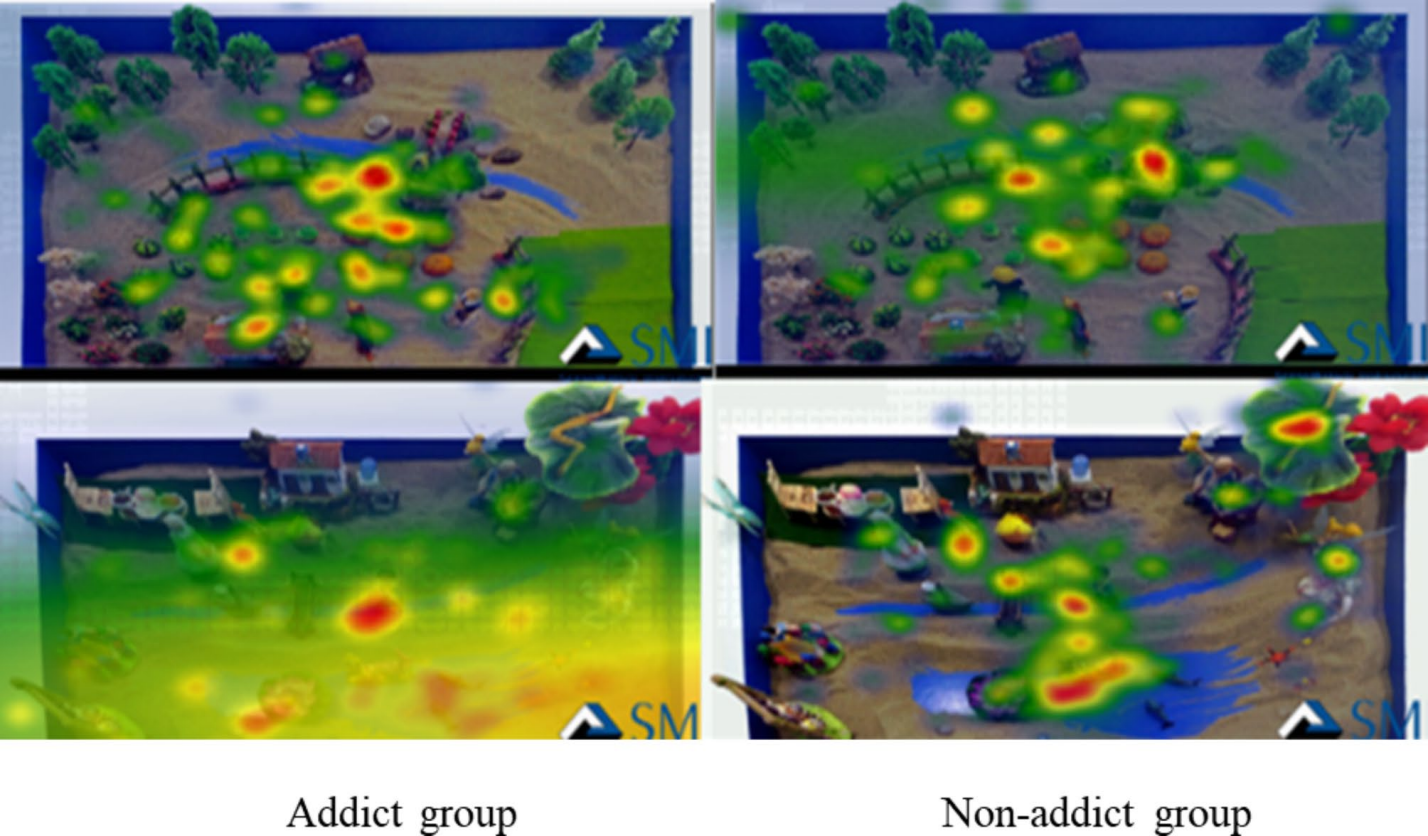
Eye Movement Heat Maps of Two Groups during Fixation on Initial Sandplay Pictures for Non-internet Addicts

As shown in Fig. 5, there was a significant difference in the eye movement heat maps between the two subject groups during fixation on the initial sandplay pictures for non-internet addicts. The addict group focused more on sand other than sand tools in the pictures, indicating a large-area fixation search.

## Discussion

The adolescent internet addict group preferred the initial sandplay pictures for internet addicts, and did not differ from the non-addict group in terms of response time.

### Behavioral data

According to the behavioral data on eye movements, the interaction and main effect of subject type and picture type were insignificant in terms of response time. The two subject groups had no difference in response time, indicating that both groups functioned normally in simple cognition and judgment. Both could make effective judgments and choices within the specified time. In terms of preference selection, the addict group preferred the initial sandplay pictures for internet addicts, indicating that the addict group had an implicit preference for things with the same characteristics as themselves [35] Thus, the initial sandplay pictures for addicts projected and presented the characteristics and subconscious content of addicts [36].

Sandplay pictures for addicts had a higher level of emotional arousal and cognitive resonance for addicts since the addicts were more familiar with such pictures and could easily process them. The same trend can also be derived from the track and heat maps.

### Eye movement indicator data

When the visual organ is diseased, it will affect the function of the eyeball, optic nerve and brain, resulting in the inability to perform normal visual activities [37]. Current studies have shown that Internet addiction may cause eye fatigue, dry eye, myopia, and other problems [38, 39]. Nevertheless, the visual function parameters and visual physiological indicators of addicts are not significantly different from those of ordinary people, and the visual organs do not appear as apparent lesions so that they can carry out normal visual activities [40]. Indeed, the visual attention processing of Internet addicts has its traits and preferences: in the face of web-related visual stimuli, addicts demonstrate higher attention preferences and search efficiency [41]. Therefore, it is practical and feasible to apply the eye tracking method to evaluate the screening and identification function of the initial sandplay picture system for internet addiction symptoms of adolescent internet addicts.Recognition of eye movements is a strategy to test the cognitive processing ability of individual subjects. Fixation duration is the average time for each fixation [42]. According to the current results, there were insignificant effects for the interaction and main effects involving subject type and picture type. This indicates that the two subject groups did not differ significantly in time allocation of attention resources during a limited period of time, possibly due to the same attentional habits.

The fixation count refers to the number of all fixations in a certain region. The number of saccades refers to the number of eye movements from one fixation to another one, and it is positively correlated with the number of fixations [42]. The number of fixations and the number of saccades is indicators of an activation effect. Thus, they determine the internal arousal state of an individual’s cognitive processing – the higher the activation level, the more information that is obtained and stored, the stronger the search capability, and the more efficient the information processing [43]. In this research, the addict group had higher numbers of fixations and saccades compared to the non-addict group, indicating that the sandplay pictures for internet addicts had a higher level of internal activation and arousal for the addict group than that of the non-addict group. Loftus and Mackworth [44] found that the subjects fixated earlier and more frequently on informative regions. The initial sandplay pictures are content-rich and informative as a whole. The extensive visual processing of regions of interest by addicts shows that the sandplay pictures for addicts have a higher level of emotional arousal and cognitive resonance.

The latency of saccades is the time between the onset of a target stimulus and the beginning of the saccade – the longer the latency of a saccade, the greater the difficulty in processing and recognizing the current target [42]. According to the current results, the latency of saccades for the adolescent internet addict group was shorter than the non-addict group during the processing of the sandplay pictures for addicts. Some other research findings have concluded that internet experience can facilitate an individual’s cognitive functioning to a certain extent. Subjects with tendencies toward internet addiction have shorter search response times compared to subjects in a relatively normal group [41]. In many cases, adolescent internet addicts have been addicted to the Internet for a long time. With rich internet experience, they are highly responsive to the processing of relevant targets. As a result, they have a shorter latency for saccades than non-addicts. They can easily search and recognize initial sandplay pictures for internet addicts since they are more familiar with such pictures and can easily process them.

### Analysis of eye movement track maps and heat maps

The track maps show the subjects’ viewing sequence of pictures and number of fixations [42]. In this research, the number of fixations of the addicts on the initial sandplay pictures for internet addicts was clearly greater than that of the non-addicts, and the fixations of the addicts focused more on conflicts and confrontations. These results were consistent with the results of the eye movement indicators (e.g., number of fixations and number of saccades). In addition, the non-addict group had more long-time fixations on the initial sandplay pictures for internet addicts, consistent with the results of the latency of saccades. The track maps show that the sandplay pictures for addicts produce high arousal for adolescent internet addicts. The heat maps show the areas with higher attention and the largest number of fixations – the redder these areas are, the more fixations there are [42]. In the process of viewing the sandplay pictures for addicts, the addict group paid more attention to conflicts, violence, threats, and isolation. These images reflect the characteristics of the initial sandplay for internet addicts (more military conflicting sand tools, and more themes on traumatic division and confusion).

## Conclusions

In conclusion, by using an eye tracking analysis and extracting participants’ visual and physiological indicators, the ecological validity and consistency of these strategies have been verified – ecological validity of the initial sandplay picture system for internet addiction symptoms in Chinese adolescents, and the consistency between the evaluation results based on sandplay pictures and those based on the Adolescent Pathological Internet Use Scale (APIUS). In turn, this supports the effectiveness and objectivity of the sandplay picture system in preliminarily screening and identifying adolescent internet addicts. But since the research is an exploratory study, the conclusions should be replicated and verified.

## Electronic supplementary material

Below is the link to the electronic supplementary material.

Supplementary Material 1

## Data Availability

The datasets used and analyzed during the current study are available from Ying Ge & Jun-yu Huo on reasonable request.
