# Supplementary material for: Eye movement verification and evaluation of initial sandplay picture system for internet addiction symptoms in Chinese adolescents
Source: Ital J Pediatr. 2023 Jul 16;49:86. doi: 10.1186/s13052-023-01487-8 (PMC10350258; doi:10.1186/s13052-023-01487-8)
Supplement: Supplementary file 1 — Supplementary Material 1 [file 13052_2023_1487_MOESM1_ESM.docx]

Dear Editor,

Thank you for your time and consideration about our manuscript.

We re-organized our manuscript and made other changes. Our changes are in red font.

Below, we list those concerns along with statements about how we updated the manuscript.

**Our Responses to Reviewer #1**

**Comment**: it is well known that patients with epilepsy may present with involvement of the eye and/or mimic muscles with more or less severe pictures. Many of these patients may also present with comorbidities such as attention deficit. What might be the 'use of this method in this setting? It would be appropriate to indicate this in the discussion.

I recommend enriching the discussion with the following article:

Unilateral Eye Blinking Arising From the Ictal Ipsilateral Occipital Area. Clin EEG Neurosci. 2016 Jul;47(3):243-6. doi: 10.1177/1550059414533111. Epub 2014 Sep 1.

**[Reply and Actions]**

Thanks. We have revised it and added relevant content in discuss section.

**Our Responses to Reviewer #2**

the manuscript is well written but needs some major revisions and in particular.

**Comment 1**: introduction is to much extended and should be shrunken and redirected to paper's aim. **[Reply and Actions]**

Thanks. We have revised them. See the manuscript in introduction section.

**Comment 2**: the result section should be better sublassified up to sub results.

**[Reply and Actions]**

Thanks. We have reclassified and renamed them. See the manuscript in results section.

**Comment 3**: discussion only addressed to results study.

**[Reply and Actions]**

Thanks. We have matched the discussion with the results in order and content. See the manuscript in discussion section.

**Other modifications**

1. We have made some modifications in materials section and discussion section.

2. References have been added, deleted and reordered.
